# Localized Induction Heating of Cu-Sn Layers for Rapid Solid-Liquid Interdiffusion Bonding Based on Miniaturized Coils

**DOI:** 10.3390/mi13081307

**Published:** 2022-08-12

**Authors:** Christian Hofmann, Maulik Satwara, Martin Kroll, Sushant Panhale, Patrick Rochala, Maik Wiemer, Karla Hiller, Harald Kuhn

**Affiliations:** 1 Fraunhofer Institute for Electronic Nano Systems ENAS, 09126 Chemnitz, Germany; 2 Center for Microtechnologies, Chemnitz University of Technology, 09126 Chemnitz, Germany; 3 Institute for Machine Tools and Production Processes, Chemnitz University of Technology, 09126 Chemnitz, Germany

**Keywords:** MEMS packaging, low-temperature bonding, selective bonding, SLID bonding, IMC, intermetallic compound, induction heating, localized heating, micro coil, electromagnetic (EM) field

## Abstract

Considering the demand for low temperature bonding in 3D integration and packaging of microelectronic or micromechanical components, this paper presents the development and application of an innovative inductive heating system using micro coils for rapid Cu-Sn solid-liquid interdiffusion (SLID) bonding at chip-level. The design and optimization of the micro coil as well as the analysis of the heating process were carried out by means of finite element method (FEM). The micro coil is a composite material of an aluminum nitride (AlN) carrier substrate and embedded metallic coil conductors. The conductive coil geometry is generated by electroplating of 500 µm thick copper into the AlN carrier. By using the aforementioned micro coil for inductive Cu-Sn SLID bonding, a complete transformation into the thermodynamic stable ε-phase Cu_3_Sn with an average shear strength of 45.1 N/mm^2^ could be achieved in 130 s by applying a bond pressure of 3 MPa. In comparison to conventional bonding methods using conduction-based global heating, the presented inductive bonding approach is characterized by combining very high heating rates of about 180 K/s as well as localized heating and efficient cooling of the bond structures. In future, the technology will open new opportunities in the field of wafer-level bonding.

## 1. Introduction

The last decades have shown an astonishing growth in the complexity of microsystems to fulfil the increasing demand of new functionalities and higher integration density. Current trends in microsystems packaging focus on the heterogeneous integration of new innovative material systems with different thermomechanical properties for the interaction of several functionalities in one smart system—so called micro-electro-mechanical systems (MEMS) [1]. Another driving force is 3D integration by stacking microsystems and integrated microelectronic components or devices on top of each other. This enables, for example, vertical interconnections with low parasitic capacitance and inductance [1,2,3]. However, a robust and hermetically sealed bond package is required to ensure reliability and functionality, especially under harsh environmental conditions, and determines the long-term drift characteristics of the bonded device. 

The hermetic encapsulation of MEMS can be realized by zero-level or wafer-level packaging techniques to combine two or more substrates with various functional components [4]. Conventional wafer bonding methods such as silicon direct bonding, anodic bonding, glass frit bonding, thermo-compression bonding, or hybrid bonding are carried out at elevated temperatures and, in some cases, with subsequent annealing of the substrates [4,5,6,7,8,9,10,11]. This can cause damage to fragile and temperature-sensitive elements. To avoid such thermal stress within the package, the bonding process of heterogeneous material stacks requires a reduction of the thermal load.

From a wide variety of bonding techniques, the SLID bonding is of great importance today and can meet these thermal requirements. The process results in hermetically sealed and mechanically stable packages with the possibility for fine-pitch bonding [12,13]. The main process advantages are moderate processing temperatures, low stresses in the final assembly and high re-melting temperatures of the formed intermetallic compounds (IMCs) [14,15]. In 1966, Bernstein presented for the first time the SLID mechanism of the binary systems Ag-In, Au-In, and Cu-In [16]. Today, Au-Sn [14,17,18] and Cu-Sn [14,19,20] are the most studied SLID systems with process temperatures above 278 °C and 232 °C, and IMC melting temperatures at about 500 °C and 700 °C, respectively. Therefore, SLID bonding is one of the most interesting low-temperature packaging technique, especially for wafer-level and 3D integration.

However, all of the above-mentioned bonding methods, including SLID bonding, are performed using a global heating of all substrates and components. A promising approach to minimize this problem is to focus the bonding temperature locally to the bond interface. Therefore, bonding processes utilizing selective heating of the interface become more relevant. One possibility to implement this approach is the use of self-propagating exothermic reactions (SER). The first application of such chemical reactions dates back to 1895, when Goldschmidt used self-propagating thermite reactions in a powder composite of aluminum and iron oxide to join railroad tracks [21]. In 1988, Clevenger et al. published exothermic Ni/Si foils, which consist of nanoscale reactive multilayer systems (RMS) [22]. Integrated reactive material systems (iRMS) represent a further development of the RMS foils. Braeuer et al. introduced direct deposition and patterning techniques of different reactive multilayer systems (Al/Pd, Al/Ti, Ti/Si) with the help of conventionally used process steps in microelectronics and microsystems technology [23,24,25]. Other publications on iRMS with integrated CuO/Al systems followed in 2018, 2020, and 2021 [26,27,28]. However, reactive bonding with iRMS requires a complex fabrication process and leads to material limitations. The deposition of the multilayers is highly time-consuming and minor deviations in the layer thickness can result in incomplete reactions.

Another very promising approach is the induction heating technology, which allows contactless transfer of highly concentrated energy into electrically conductive materials with a high degree of efficiency [29,30]. However, current research work on induction heating is mainly concentrated in macroscopic fabrication processes. With inductive contact joining (ICJ), metals and composites can be bonded very efficiently [31,32]. Another field of induction-based joining is the longitudinal welding of pipes from advanced high strength steels (AHSS) by high-frequency induction welding. The authors demonstrated the longitudinal welding of 42SiCr- [33] and 34MnB5-pipes [34] in combination with subsequent heat treatments to manufacture pipes with enhanced mechanical properties. 

In 2002, induction heating for bonding at microscopic level was used for the first time by Thompson et al. to support silicon direct bonding with subsequent inductive annealing of the substrates [35]. Since 2002, the number of publications on inductive heating for MEMS packaging with ferromagnetic and solder materials (e.g., Ni/Co, Sn/Pb) has increased gradually [36,37]. Chen et al. investigated the correlations of induction heating between pattern design on printed circuit boards and determined the heat distribution by FEM and infrared (IR) thermal imager [38]. However, due to the structural dimensions as well as material combinations, the integration into microtechnological and industrial manufacturing chains could not be demonstrated. In the previous work of the authors in 2019 and 2020 [27,39], a method for selective induction heating of Cu-Sn layers for energy-efficient bonding at wafer-level were realized for the first time, using an optimized coil design. In order to heat industrially relevant bond structures with sufficient efficiency and homogeneity, the induction coils need to be miniaturized continuously. This would enable specific integration into economic manufacturing processes. 

Meanwhile, Yin et al. [40] and Sun et al. [41] demonstrated that inductive heating of the binary system Cu-Sn leads to significantly faster intermetallic phase formation of Cu_6_Sn_5_ and Cu_3_Sn. Moreover, Hammad et al. was able to achieve significantly improved microstructure features and mechanical properties of inductively heated tin-silver-zinc alloys after IMC formation [42]. Furthermore, induction heating can be used for sintering of micro-scaled silver particles to bond microelectronic components at chip-level [43]. 

In the present paper, FEM, design, and fabrication of a novel miniaturized induction coil based on a ceramic-metal compound for inductive Cu-Sn SLID bonding is proposed. The coil design is adapted to the bond layout for homogeneous and rapid heat generation. Additionally, a diffusion model for the binary system Cu-Sn could be developed using FEM, which provides a correlation between layer thickness, temperature, and time as well as characteristic details of the formed IMCs during the bonding process. Furthermore, the approach, design, fabrication, and assembly of an inductive bonding module are described in detail. In comparison to the previous work [27,39], bond structures with considerably smaller lateral widths were used during the experiments. The heat-treated and bonded structures were characterized in terms of temperature distribution, heating rate, microstructure, and mechanical strength. 

## 2. Physical Background

### 2.1. Induction Heating

According to Ampere’s law, an electric current flow through a primary conductor (here referred to the micro coil) forms an alternating EM field around the coil. By defined positioning of a secondary conductor (here referred to the electrically conductive Cu-Sn bond layer) in the EM field, an induced voltage *U_i_* generates eddy currents *I* inside the conductor. Due to the ohmic resistance *R* of the secondary conductor material, the eddy currents cause a heat flow ${\dot{Q}}_{el}$ based on the Joule effect, which is locally limited to the area where the magnetic field penetrates the material. Equation (1) determines the heat flow in the secondary conductor with the cross-section *A*.

(1) $${\dot{Q}}_{el}=I^{2}\cdot R=\int_{A}J^{2}dA\cdot R$$

The heat concentration at the secondary conductor surface increases exponentially with increasing frequency *f*_0_ and can be defined by the current density *J*(*x*) (2).

(2) $$J\left(x\right)=J_{0}\cdot e^{\left(-\frac{x}{\delta }\right)}$$

Depending on the frequency, the alternating eddy current in the secondary conductor tends to move to the outer surface, which reduces the internal magnetic fields in the material. The current density *J*_0_ is maximum on the surface, dropping off exponentially toward the center of the secondary conductor due to the skin effect. The skin depth *δ* (3) determines the skin effect and represents the distance from the secondary conductor’s surface where the current density is reduced to 37% of its maximum value. In this area, about 86% of the electric energy is converted into heat [29,30].

(3) $$\delta =\frac{1}{\pi \cdot f_{0}\cdot \mu \cdot \sigma }$$

The skin depth depends on the permeability *μ*, the electrical conductivity *σ*, and the frequency *f*_0_ of the EM field. Table 1 shows the dependence of the skin depth for copper and tin as a function of the frequency in the range of 1 MHz to 2 MHz.

Due to the edge effect, the majority of the induced current density is concentrated at the border of the heated material, which leads to inhomogeneous temperature distribution. To avoid such phenomenon, the coil size should approximately match the size of the structure to be heated. The edge effect can be neglected for the heating of micro-scaled layers considering the scale and conductivity of the material [44].

In addition to the skin effect, energy transmission due to EM heating also depends on the coil geometry, the distance between coil and bond layer (here referred to coupling distance), input power, heating time, conduction, convention, radiation, and temperature-dependent properties of the material. To increase the EM heating efficiency, it is important to adapt the coil geometry to the geometrical dimensions of the heated structure [45]. 

**Figure 1:**
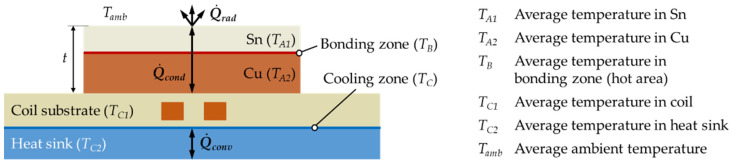
Illustration of the conventional heat transfer modes convection, conduction, and radiation using the example of a setup with two bond partners, a coil substrate, and a heat sink.

Figure 1 shows a schematic representation with considered heat losses based on an exemplary setup consisting of coil conductors embedded in a substrate, a water-cooled heat sink, and two bond partners (copper and tin). The traditional transfer modes for heat energy with reference to convection, conduction, and radiation can be used in various combinations to generate heat and transport it through the individual layers as well as components. In contrast to resistive-based heating, where heat propagates from the source to the bonding zone via a variable mix of heat transfer modes, induction-based heating can generate the desired heat in the bonding zone itself.

In the illustrated example, the heat generated in the material is dissipated by convection ${\dot{Q}}_{conv}$ from the coil substrate through the heat sink (4), by conduction ${\dot{Q}}_{cond}$ between the bond partner copper and tin (5), and by radiation ${\dot{Q}}_{rad}$ from the tin surface (6).

(4) $${\dot{Q}}_{conv}=h\cdot A_{C}\cdot \left(T_{C1}-T_{C2}\right)$$

(5) $${\dot{Q}}_{cond}=\frac{A_{B}\cdot \lambda }{t}\left(T_{B}-T_{A1|A2}\right)$$

(6) $${\dot{Q}}_{rad}=\varepsilon \cdot \sigma \cdot A_{A1}\cdot \left(T_{A1}^{4}-T_{amb}^{4}\right)$$

The different values are related to the components of the setup, where *h* is the heat transfer coefficient, *λ* is the thermal conductivity, *t* is the total thickness of the bond partner, *ε* is the emissivity, and *σ* is the Stefan–Boltzmann constant. *A_C_* refer to the area of the cooling zone, *A_B_* to the area of the bonding zone, and *A_A_*_1_ to the area of the tin surface.

### 2.2. Solid-Liquid Interdiffusion Bonding

The SLID bonding (also known as Transient Liquid Phase Bonding) is a packaging technique based on intermetallic diffusion between two or more metals, with a high-temperature melting metal (such as Cu or Au) and a low-temperature melting metal (such as Sn or In) under the influence of temperature, time, and pressure. Intermetallic formation is driven by the atomic concentration gradient and can be described by the second Fick’s law of diffusion [46]. It states that the diffusive flux goes from a high-concentration area to a low-concentration area proportional to the concentration gradient. According to the Arrhenius Equation (7), the diffusivity is determined by the diffusion coefficient *D*_0_, the activation energy *E_A_*, the universal gas constant *R*, and the absolute temperature *T*.

(7) $$D=D_{0}\cdot \exp\left(-\frac{E_{A}}{RT}\right)$$

**Figure 2:**
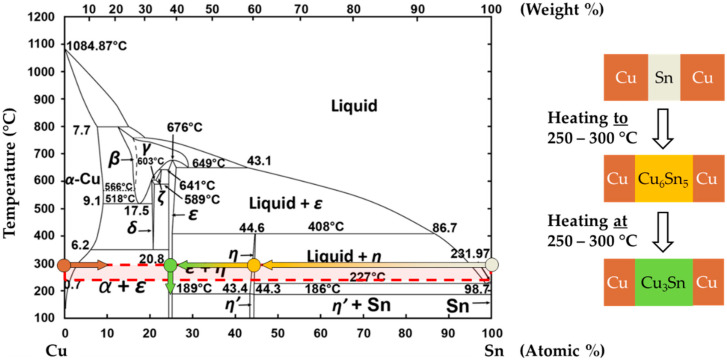
Representation of the Cu-Sn phase diagram with the IMC formation, where the red dot and gray dot are related to the original copper and tin layers, respectively. The yellow dot shows the first formed η-phase Cu_6_Sn_5_, the green dot the stable ε-phase Cu_3_Sn. Reprinted/adapted with permission from Ref. [48]. Copyright 2017, copyright Charlie Sanabria.

Figure 2 illustrates the Cu-Sn phase diagram with the IMC formation based on the temperature and the relative material concentrations. The diffusion process of unalloyed copper and tin will accelerate by exceeding the melting point of Sn (*T_M_Sn_* = 232 °C). The temperature range used in the literature is between 250 °C and 300 °C [14,15,19,20,46], resulting in intermetallic bonds of Cu_6_Sn_5_ (η-phase) and Cu_3_Sn (ε-phase) during the bonding process. The η-phase is formed in the early heating stage. As the interdiffusion continues, the ε-phase starts to grow at the Cu_6_Sn_5_/Cu interface. Compared to the metastable η-phase, the thermodynamically stable ε-phase possesses significantly improved thermal and mechanical properties. In order to obtain the preferred ε-phase, the binary system should be designed in such a way that the Cu can absorb most of the Sn [47]. Since a non-uniform heat input can lead to voids and inclusions during the diffusion process, it is also important to achieve homogeneous temperature distribution in the bonding area.

Based on the fundamental findings of Yin et al. [40] and Sun et al. [41], the diffusion rate of Sn at temperatures above its melting point is accelerated and leads to Cu_3_Sn phases with increased thicknesses. Due to the highly selective and very fast inductive heat input, it is possible to overheat the material system locally. As a result, the diffusion process can be expedited significantly, without majorly affecting the entire bonding substrates.

## 3. Experimental Setup

### 3.1. Design and Materials

**Figure 3:**
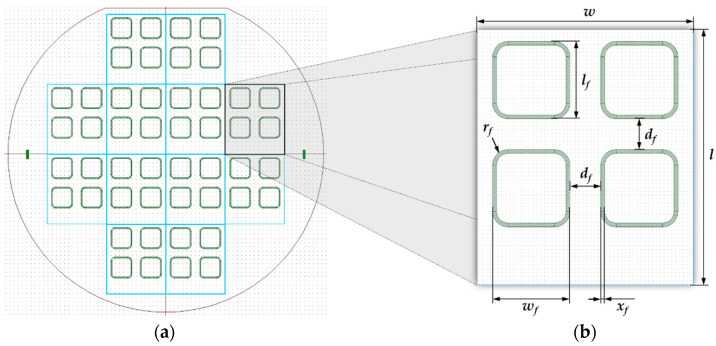
(**a**) Wafer design with twelve symmetrically arranged single chips; (**b**) Single chip with four bond frames.

For performing the inductive bonding experiments, heterogeneous chip-level stacks were used in this study as the joining partner. The substrate materials include a combination of silicon and Borofloat^®^ 33 glass with thicknesses of *h_Si_* = 675 µm and *h_G_* = 500 µm, respectively (SCHOTT Technical Glass Solutions GmbH, Jena, Germany). A 6-inch wafer was structured in twelve bond chips, which are shown in Figure 3. Each chip contains four bond frames on an area of 33 × 28 mm^2^. The frames served as test structures to validate the prospective hermetic encapsulation of MEMS devices at wafer-level.

Due to the comparatively high thermal conductivity of silicon with approximately *λ_Si_* = 150 W/(m·K) [49,50], silicon dioxide (SiO_2_) layers with a thickness of *h_SiO2_* = 2 µm were deposited on the wafer surface, which act as a thermal barrier to partially compensate the heat dissipation during inductive heating. An overview of the most relevant material and layout parameter of the bond substrates is given in Table 2.

The deposition of the Cu-Sn frames onto the substrates was performed by electroplating (electrochemical deposition—ECD) of 1.0 µm thick tin and 2.5 µm thick copper. The geometrical and material parameters of the bond frames are listed in Table 3. 

**Figure 4:**
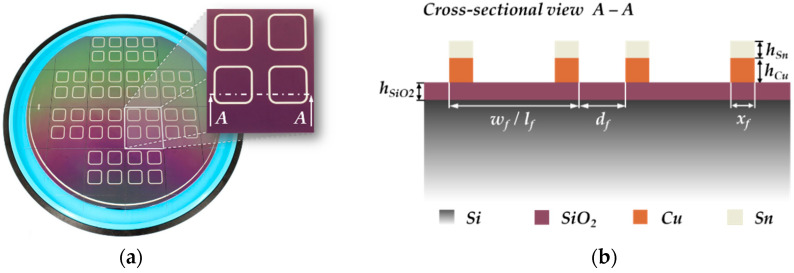
Bond substrates after fabrication: (**a**) 6-inch silicon wafer after dicing in 12 single chips; (**b**) Cross-sectional view of two Cu-Sn frames.

The most important parameter from Table 3 is the lateral frame width. This value essentially determines the width of the corresponding micro coil, and thus, the cross section and thermal behavior of the coil during the process. After the ECD, the electrically conductive seed layer was removed. Finally, the wafer could be separated into single chips. Figure 4 shows a diced silicon wafer with a cross section of the Cu-Sn frames.

### 3.2. Simulation Model

**Figure 5:**
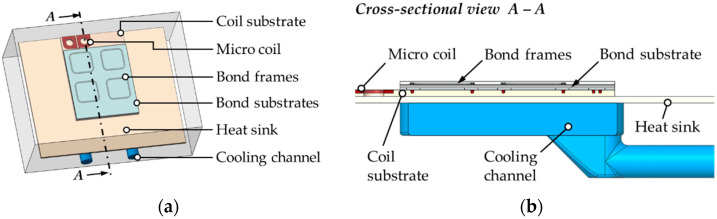
Simulation model with the major components: (**a**) Top view; (**b**) Cross-sectional view.

A frequency-transient study in COMSOL Multiphysics^®^ was applied to determine the EM heating in the metal frames, the water-cooling based on convection (4) and the heat losses due to the heat transfer modes conduction (5) and radiation (6). The simulation model in Figure 5 represents the basis of the mentioned physical mechanism.

The model contains the exact geometries of the micro coil and the bond setup as well as simplified components of the inductive bonding module (cooling channel, heat sink, thermal isolators, and ambient air). The physics-controlled meshing was performed using tetrahedra and swept elements to fit the mesh size to the respective geometry. To perform the analysis, the AC/DC module, the heat transfer module, and detailed material parameter from the software database were used. Simplifications were made for the thermal and electrical contact points, resulting in the assumption of an ideal surface transition. Furthermore, the simulation model relies on temperature-dependent material properties such as thermal conductivity, specific heat capacity, volume resistivity, thermal expansion coefficient, and density. In addition, the radiation effect for each material could be determined by assigning the emissivity in the boundary conditions. In order to consider the cooling of the micro coil and the overall system, the electromagnetic and thermal simulations were combined with the consideration of a laminar flow. In the boundary conditions, a water flow velocity of *v* = 0.66 m/s was specified, which corresponds to a water flow rate of *Q* = 1 L/min including the channel cross section. The water temperature within the channels was set to *T_W_* = 20 °C. The aim was to geometrically design the cooling channel without turbulences (e.g., vorticity, cross flow) and to efficiently cool the micro coil. During the FEM, electrical parameters (coil current *I*_0_, frequency *f*_0_), the heating time *t_h_*, the coupling distance *d*, and harmonics could be varied.

### 3.3. Micro Coil

**Figure 6:**
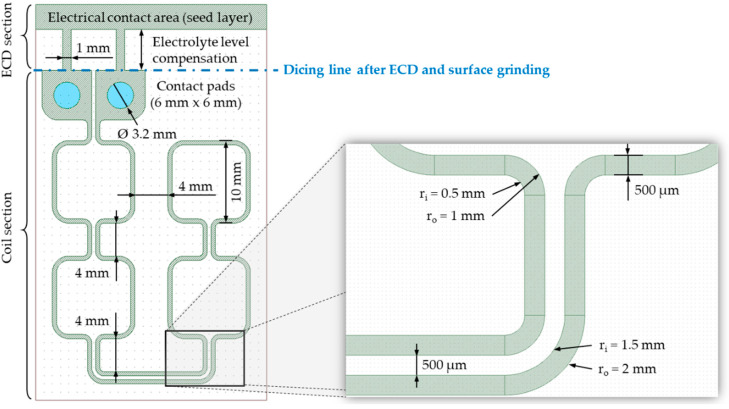
Micro coil design with the ECD section as well as the coil section.

The definition of the coil layout (Figure 6) was based on the simulation results of the coil geometry (Section 4.1). In order to ensure an accurate fabrication process, the layout consists of two connected sections:

- ECD section with electrically conductive contact area for electroplating as well as electrolyte level compensation;
- Coil section with conductor lines as well as contact pads for smart connectivity with the induction generator.

Aluminum nitride (AlN, CeramTec Alunit^®^ 170 C) was used as substrate and carrier material for the micro coil fabrication. Due to its excellent thermal conductivity, high volume resistivity, and high dielectric strength, AlN fulfills the relevant requirements for the application as coil substrate. The AlN parameters are listed in Table 4.

**Figure 7:**
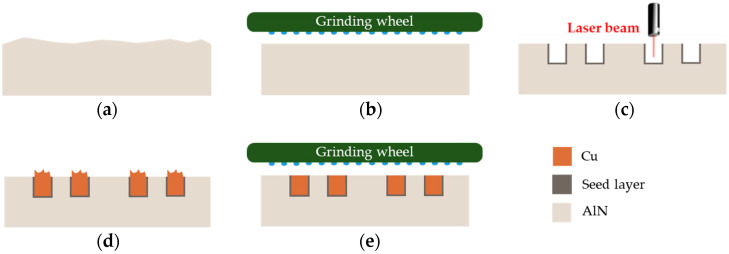
Schematic process flow for micro coil manufacturing with integrated copper conductors: (**a**) Carrier substrate (as fired surface); (**b**) Surface finish; (**c**) Laser micromachining; (**d**) Copper ECD; (**e**) Coarse and fine surface grinding of the copper outgrowth and the AlN surface.

The process flow for the integration of miniaturized and metallic coil conductors into an AlN carrier substrate consists of several process steps (Figure 7). First, the surface quality of the carrier substrate needs to be improved to ensure suitable surface roughness and flatness for subsequent process steps and the micro coil application for induction heating. Subsequently, laser micromachining is performed to generate the coil geometry as well as the electrically conductive seed layer. The seed layer is used to fill the laser-patterned AlN trenches using copper ECD. Finally, the copper outgrowth generated during the ECD process is removed by a coarse and fine grinding step.

**Figure 8:**
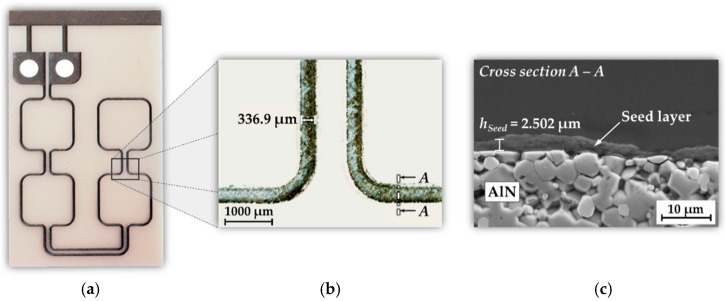
AlN carrier after laser processing: (**a**) Photographic image of the coil substrate; (**b**) Macroscopic image of laser-patterned coil conductors (3.0× magnification); (**c**) SEM cross section of the laser-generated seed layer on top of AlN.

After surface finishing, the AlN carrier substrate can be patterned by laser processing. The most important requirements for the process are the lateral conductor width, the trench depth, and the roughness of the trench side walls. Furthermore, the thermal stress of the substrate during laser patterning should be minimized to prevent cracks in the material and chipping of the conductor lines. Based on the simulation of the coil geometry (Section 4.1), an optimized conductor cross section could be determined. A lateral conductor width of *w_c_* = 500 µm with a similar conductor thickness of *h_c_* = 500 µm results in good inductive heating efficiency, acceptable self-heating of the coil conductors, and the best possible manufacturing feasibility of the micro coil in terms of laser structuring and Cu ECD. Due to the significantly higher material removal rate, the deep laser ablation for patterning the basic coil geometry was realized with the ultrashort pulse laser. Afterwards, the pre-patterned substrate was post-processed with the nanosecond laser to generate the electrically conductive seed layer. The laser pulse leads to an ionization of the upper atomic AlN substrate layer and consequently to the formation of a hot, dense plasma. The resulting reduction of AlN to aluminum, nitrogen and additional atomic constituents forms an oxidation-resistant and electrically conductive intermetallic compound on the laser-machined surface. The sheet resistance was determined with the 4-point probes method [53] and is in the range of *R_Seed_* = 3.2 to 6.9 Ω. The thickness of the generated seed layer is in the range of 1 µm to 3 µm, depending on the surface condition and the laser pattern. Figure 8 shows the AlN substrate after laser processing of the ECD section as well as the coil section.

The macroscopic image shows a lateral conductor width of *w_t_* ≈ 330 µm at the laser-patterned trench end. Consequently, the ablation process results for the used laser setup in a depth-dependent conicity of 1/3. Measurements with confocal microscopy indicated a slope angle of approximately 83°. With the pre-defined lateral coil conductor width of *w_c_* = 500 µm at the surface of the AlN substrate as well as a trench depth or conductor thickness of *h_c_* = 500 µm, the trench width *w_t_* can be determined with (8).

(8) $$w_{t}=w_{c}-\left(\frac{h_{c}}{3}\right)$$

The volume resistivity *ρ_Seed_* can be calculated from the sheet resistance *R_Seed_* and the seed layer thickness *h_Seed_* using the following Equation (9).

(9) $$\rho_{Seed}=h_{Seed}\cdot R_{Seed}$$

Assuming a 2.5 µm thick seed layer, the resulting volume resistivity is in the range of *ρ_Seed_* = 8.0 × 10^−4^ Ω·cm to 1.73 × 10^−3^ Ω·cm.

**Figure 9:**
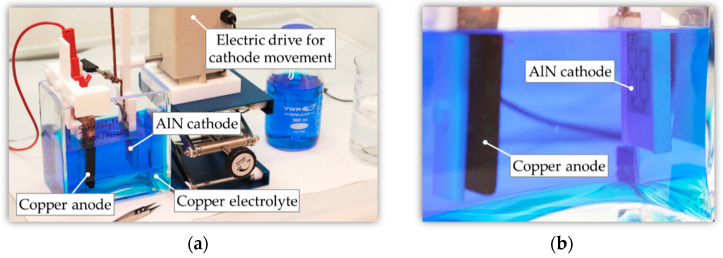
Copper ECD of the laser-patterned AlN substrates: (**a**) Full setup with beaker, electrolyte, anode, cathode, and electric motor; (**b**) Arrangement of copper anode and AlN cathode.

The subsequent copper ECD was performed using an experimental beaker setup (Figure 9). The electrical connection of the AlN substrate (cathode) was realized onto the seed layer of the ECD section by means of a crocodile clip. Prior to the deposition process, a pre-treatment of the laser-machined AlN substrates was carried out. The substrates were cleaned in DI water and isopropanol with ultrasonic assistance to remove the residual dust layer caused by the laser process. Subsequently, the deposition was performed in a two-step process sequence with current densities in the range of *J* = 4 A/dm^2^ to 6 A/dm^2^ for *t* = 450 min. The first deposition step was used to increase the thickness of the thin and sensitive intermetallic seed layer. In the second step, the coil trenches were completely filled. The aim was to overgrow the coil trenches with the deposited copper (Figure 7d).

The theoretical mass of the Cu layer *m_Cu_* can be determined using electrogravimetry with Faraday’s law of electrolysis (10), where *M_Cu_* is the molar mass of copper, *Q* is the electrical charge, *z* is the ionic charge, and *F* is the Faraday constant.

(10) $$m_{Cu}=\frac{M_{Cu}\cdot Q}{z\cdot F}$$

Thereafter, the thickness of the deposited copper layer *h_Cu_* can be calculated using the following Equation (11), where *ρ_Cu_* is the copper density and *A_Ca_* is the entire cathode area.

(11) $$h_{cu}=\frac{m_{Cu}}{\rho_{Cu}\cdot A_{Ca}}$$

**Figure 10:**
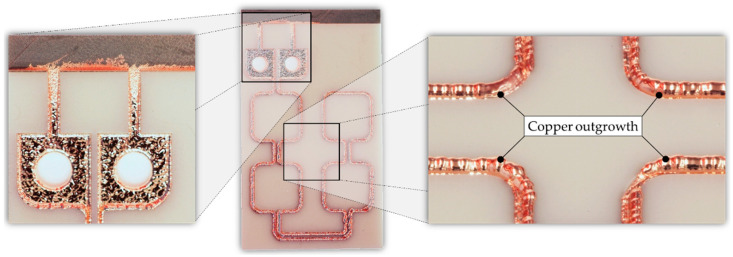
Photographic images of the AlN carrier after ECD process with copper filled trenches.

Figure 10 demonstrates an AlN substrate after the two-step process sequence with the copper outgrowth in the coil conductors. The deposited copper layer had a theoretical mass of approximately *m_Cu_* = 0.68 g and a thickness of *h_Cu_* = 603.2 µm. This corresponds to an average deposition rate of approximately *a* = 1.34 µm/min.

**Figure 11:**
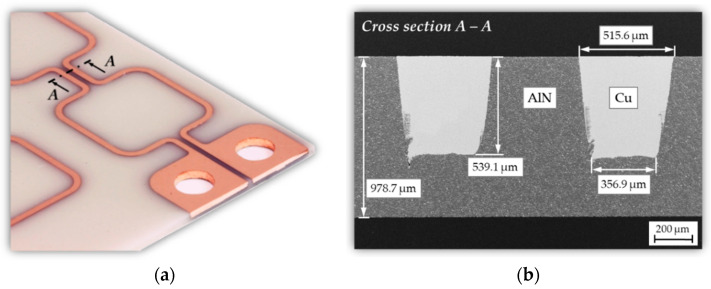
AlN/Cu micro coil after manufacturing process: (**a**) Photographic image of the micro coil; (**b**) SEM cross section of two copper conductor lines embedded in AlN.

After ECD, a subsequent grinding process was used to ensure a flat conductor surface without defects and voids. Finally, the ECD section is separated by a dicing step to remove the short circuit of the coil conductors. The final dimension of the AlN substrate is 40 mm × 28 mm. The finished micro coil substrate with the Cu conductors embedded in AlN as well as a corresponding SEM cross section are shown in Figure 11. The coil conductors have a maximum thickness of *h_c_* = 539 µm with a lateral width of *w_c_* = 515 µm on the surface and *w_c_* = 357 µm at the bottom of the trench. Finally, the electrical characteristics of the coil were determined using the impedance analyzer 16,777 k (SinePhase Instruments GmbH, Austria) for a resonance frequency of *f*_0_ = 2 MHz. The coil has an inductance of *L* = 124 nH and an electrical resistance of *R* = 0.116 Ω.

### 3.4. Inductive Bonding Module

**Figure 12:**
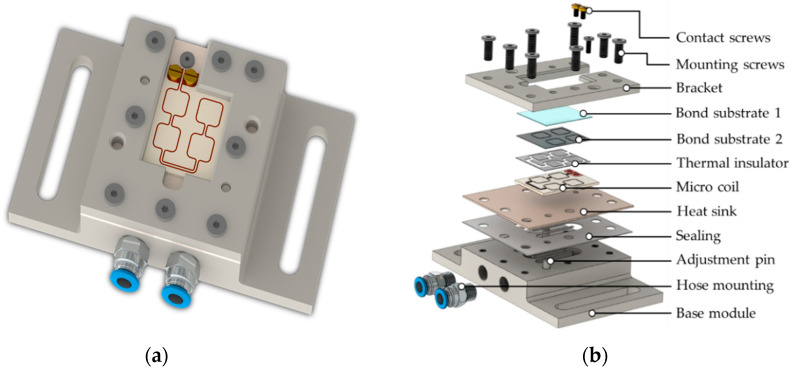
CAD modeling of the inductive bonding module: (**a**) Assembly of the module; (**b**) Exploded view of the module with important components and materials.

In order to perform the inductive heating and bonding experiments with the fabricated micro coil, the conceptual design and fabrication of a bonding module was necessary. The design required the implication of complex interactions between coil self-heating, inductive frame heating as well as the water-cooling and was based on the simulation results of the thermal management (Section 4.2). The computational design (3D CAD model) of the module with major components is presented in Figure 12.

For the dimensioning of the module, the following aspects had to be considered in order to ensure efficient inductive heating and bonding:

- Transfer of the entire experimental setup consisting of bonding module, micro coil, and bond substrates to the inductive bonding system
- Efficient cooling of the micro coil during induction heating;
- Electrical connection of the micro coil with the induction generator;
- Alignment of the bond structures to the coil layout.

**Figure 13:**
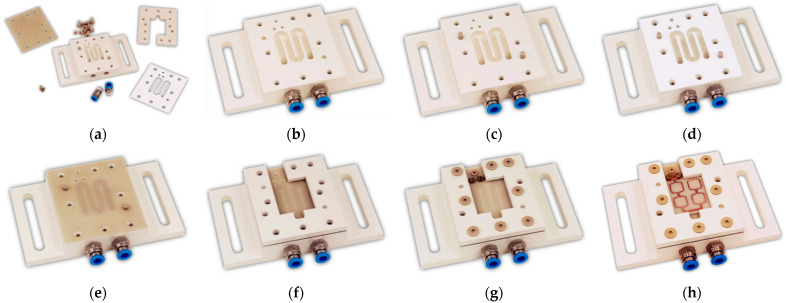
Assembly of the inductive bond module: (**a**) Individual component; (**b**) Base module with hose mounting; (**c**) Pins for component adjustment; (**d**) Water sealing; (**e**) Heat sink; (**f**) Bracket; (**g**) Screwing with mounting and contact screws; (**h**) Bonding module with Cu/AlN micro coil.

The cooling concept includes a universal cooling system consisting of a base module with standardized meander-shaped cooling channels and an unstructured cover plate as a heat sink. The base module and the bracket were made of a composite polymer with very high-volume resistivity to prevent undesired electromagnetic coupling into the module as well as improved thermal conductivity and temperature stability. The assembly of the inductive bond module is illustrated in Figure 13.

In order to ensure maximum flexibility of the bonding process and, if required, to efficiently modify the process setup (e.g., micro coil, heat sink, connection cables), a concept for the electrical supply of the micro coil was developed. This concept has to fulfill the following requirements:

- Connection concept: releasable, mechanical screw fitting without soldering;
- Connection cable: high frequency litz wire for low electrical losses;
- Connection element/fastener: cable lug, screw.

These requirements were directly adopted to the designing and fabrication of the micro coil (through holes in the coil pads) as well as the dimensioning and manufacturing of the bonding module (access points, screw threads). The result is a setup that allows the coil substrate to be attached to the bonding module and to establish the electrical contact. To prevent electrical sparks and breakdowns between the connection points, PTFE with a high dielectric breakdown strength of *E_BD_* = 20 kV/mm was used as the insulation material.

### 3.5. Inductive Heating System

Since low skin depths (3) are preferable for efficient inductive heating of very thin metal layers, the experiments were performed in high frequency range. Thus, a Sinus 102 (Himmelwerk Hoch- und Mittelfrequenzanlagen GmbH, Tübingen, Germany) induction generator with an operating frequency in the range of 1 MHz to 2 MHz, a maximum output power of *P* = 10 kW, as well as a parallel operating resonant inverter was used. The inverter adjusts the resonance frequency *f*_0_ of the current and magnetic field as well as the magnitude of the current by means of the impedance. The electric resonant circuit is in resonance when the capacitive reactance of the capacitor *X_C_* and inductive reactance of the coil *X_L_* are equal (*X_C_* = *X_L_*). Consequently, an entirely ohmic impedance (effective resistance *R*) results in the oscillating system. The coil inductance *L* and the total capacitance *C* of the inverter arrangement determine the LC resonant circuit. The resonance frequency *f*_0_ can be calculated using Thomson’s equation of oscillation (12).

(12) $$f_{0}=\frac{1}{2\pi \cdot \sqrt{L\cdot C}}$$

**Figure 14:**
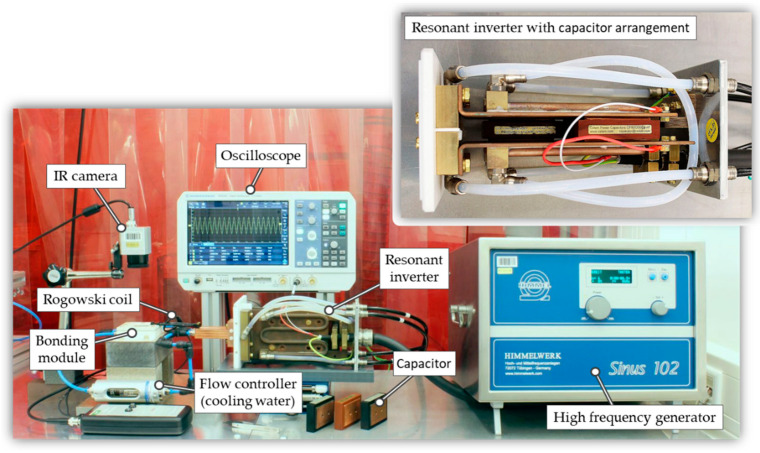
Inductive heating setup with high frequency induction generator, resonant inverter, bonding module and monitoring equipment (Rogowski coil, oscilloscope, flow controller).

Since the used coil determines the inductance, the frequency can only be changed by adjusting the capacitance of the resonant inverter. Figure 14 shows the setup for the induction heating experiments. Various measurement and control devices were used for the process monitoring. A flow controller was installed to adjust and monitor the water flow through the bonding module. To determine the resulting coil current as well as the precise resonance frequency, the Rogowski coil CWT MiniHF 6 (PEM—Power Electronic Measurements Ltd., Long Eaton, UK) was used. An amplifier and an oscilloscope realized the metrological recording of the measured data. An IR camera PI 640i (Optris GmbH, Berlin, Germany) with microscope optic was used to evaluate the bond structures in terms of heating rate and temperature homogeneity.

### 3.6. Inductive Bonding System

**Figure 15:**
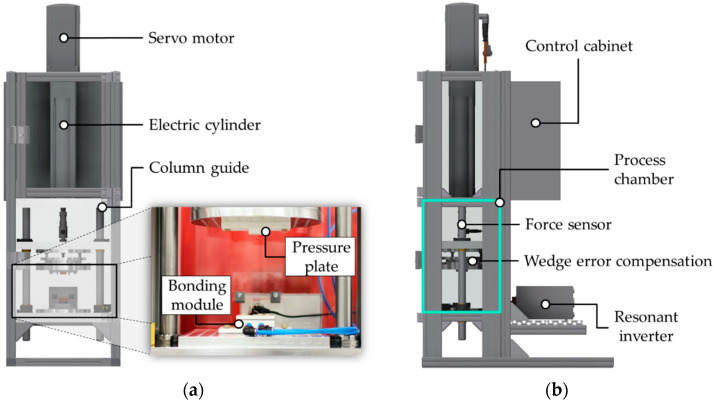
Inductive bonding system with mechanical drives, wedge error compensation, process chamber, bonding module, and resonant inverter: (**a**) Front view; (**b**) Side view.

To automatically perform the bonding experiments, an integrated system is required that includes the inductive bonding module (Section 3.4), the inductive heating system (Section 3.5), mechanical components, monitoring devices, and the control cabinet. Therefore, a concept for a bonding system was developed and realized that imitates a wafer bonder by a modular rig with improved flexibility in terms of usable units (i.e., inductive bonding module) and energy sources (i.e., inductive heating system) as well as improved accessibility for electrical cable routings and process monitoring. The computational 3D model of the entire system with the major components such as the servo motor, the column guide, and the wedge error compensation is illustrated in Figure 15. In terms of the mechanics, an adjustable and defined bonding pressure is required. Therefore, a servo motor drive (maximum pressing force of *F* = 17 kN) with an integrated force sensor (accuracy ± 0.25%, repetition accuracy ± 0.01 mm, scanning frequency 1 kHz) and a control unit was used to precisely move the bonding module and to apply specific bond pressures. The process chamber encapsulates the bonding modules and allows constant atmospheric process conditions. A combination of a column guide frame and a wedge error compensation ensures parallel applied and homogeneous bonding pressure. Furthermore, the separation of the bonding chamber, the drivetrain, and the additional units (e.g., control cabinet, inductive heating system) increases the mechanical precision.

## 4. Results and Discussion

### 4.1. FE Simulation of Coil Geometry

**Figure 16:**
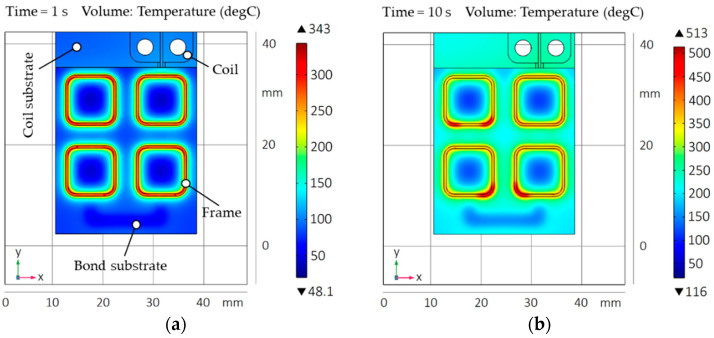
FE simulation for designing the micro coil geometry: (**a**) Bond frame and substrate temperature after *t_h_* = 1 s; (**b**) Bond frame and substrate temperature after *t_h_* = 10 s.

The aim of the geometry simulation is to evaluate a suitable micro coil design for rapid and homogeneous heat development in all metallic bond frames in a single heating step. To determine the influence of the EM field with focus on temperature distribution and heating rate, the coil was optimized in terms of basic geometry, conductor width, conductor cross section, conductor displacement, relative position of the conductors to the frames, and coupling distance of the coil to the frames. The results were the initial design data for manufacturing the micro coil (Section 3.3). The FEM was carried out with a coil current of *I*_0_ = 50 A, a frequency of *f*_0_ = 2 MHz (upper limit of the induction generator), and heating times in the range of *t_h_* = 1 s to 10 s. Borofloat^®^ 33 glass with Cu-Sn frames analogous to the specifications in Table 2 and Table 3 was used. The resulting coupling distance was *d* = 0.5 mm. Figure 16 illustrates the simulation results of the heat distribution as well as the maximum temperature in the bond substrate and frames for the finalized meander-shaped coil design (Figure 6). The simulation images compare the temperature progression after heating times of *t_h_* = 1 s (a) and *t_h_* = 10 s (b).

The simulation after one second shows a maximum temperature in the bond frames of *T_max__*_1_ = 343 °C with an average temperature of about *T_Ø__*_1_ = 271 °C as well as a minimum temperature in the bond substrate of *T_min__*_1_ = 48.1 °C. The heating in the bond frames results in a homogeneous temperature distribution. After ten seconds, the heat input into the frame corners increases to *T_max__*_10_ = 513 °C with an average frame temperature of about *T_Ø__*_10_ = 366 °C. Due to self-heating of the coil conductors (1), convection (4), and conduction (5), the minimum bond substrate temperature increases to *T_min__*_10_ = 116 °C. Thus, strictly localized heating of the bond frames (selectivity) will not be achieved without additional cooling (temperature management, Section 4.2). However, a rapid and homogeneous heat input into the bond frames could be demonstrated with the used coil geometry.

### 4.2. FE Simulation of Thermal Management

The aim of the thermal management simulation is to configure a setup for efficient cooling of the micro coil as well as high temperature selectivity between the induction-heated bond frames and the surrounding materials. Therefore, the simplified simulation model (Section 3.2) was optimized with respect to material and geometry parameters of the cooling channel as well as the heat sink. The results were the initial design data for manufacturing the inductive bonding module (Section 3.4). To compare the results with the FE simulation of the coil geometry (Section 4.1), identical electrical process parameters with *I*_0_ = 50 A and *f*_0_ = 2 MHz were used. The chosen heating times varied in the range of *t_h_* = 1 s to 10 s. The coupling distance was *d* = 0.5 mm.

**Figure 17:**
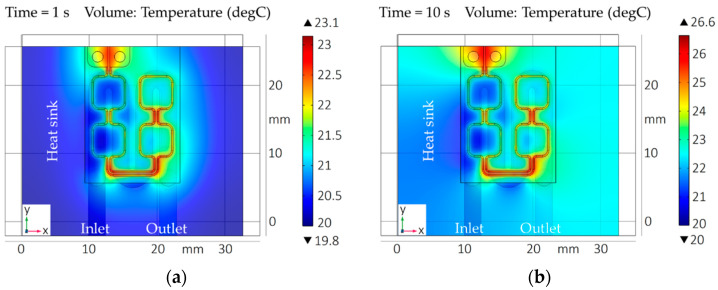
FE simulation of the thermal management based on a passively water-cooled micro coil: (**a**) Coil and heat sink temperature after *t_h_* = 1 s; (**b**) Coil and heat sink temperature after *t_h_* = 10 s.

Figure 17 shows the temperature distribution in the conductors and the coil substrate after *t_h_* = 1 s (a) and *t_h_* = 10 s (b) using an AlN heat sink with a thickness of *h* = 1.5 mm. Due to the water inlet and outlet position of the cooling channel, the water and coil temperature increase continuously towards the outlet. However, the maximum coil temperature of *T_max__*_10_ = 26.6 °C after *t_h_* = 10 s demonstrates that the temperature in the coil and surrounding components can be limited during the inductive heating process.

**Figure 18:**
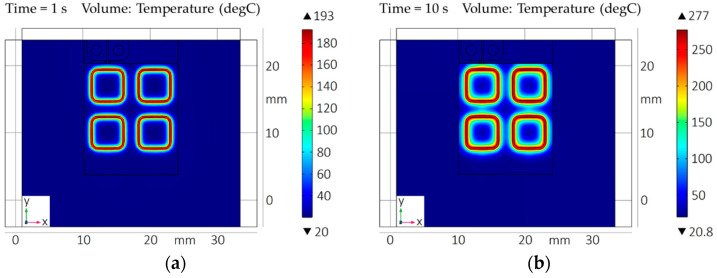
FE simulation of the thermal management for the resulting inductive heating of the bond frames in relation to the bond substrate, the micro coil, and the heat sink: (**a**) Temperature distribution after *t_h_* = 1 s; (**b**) Temperature distribution after *t_h_* = 10 s.

The resulting inductive heating of the bond frames after *t_h_* = 1 s (a) and *t_h_* = 10 s (b) is shown in Figure 18. Compared to the heating without water cooling system (Figure 16), a lower average frame temperature of *T_Ø__*_1_ = 183 °C after one second and *T_Ø__*_10_ = 263 °C after ten seconds is achieved using the cooling module. The remaining model components (micro coil, bond substrate, and heat sink) stay close to room temperature. Thus, an almost selective heating of the bond frames is achieved. Furthermore, the simulation results indicate that the average heating rate of *dT*/*dt* = 183 K/s within one second is significantly higher than the heating rate of *dT*/*dt* = 26.3 K/s within ten seconds. Accordingly, the increase in the frame temperature as a function of time will continuously decrease at constant coil current and frequency. This will lead to the formation of a temperature plateau.

### 4.3. Inductive Heating

The inductive heating system (Figure 14) was applied to characterize the heating and cooling rate as well as the temperature distribution of the entire bond setup. The aim was the validation of the FEM results and the parameterization for the inductive bonding experiments (Section 4.4) using IR thermography imaging and current measurement.

**Figure 19:**
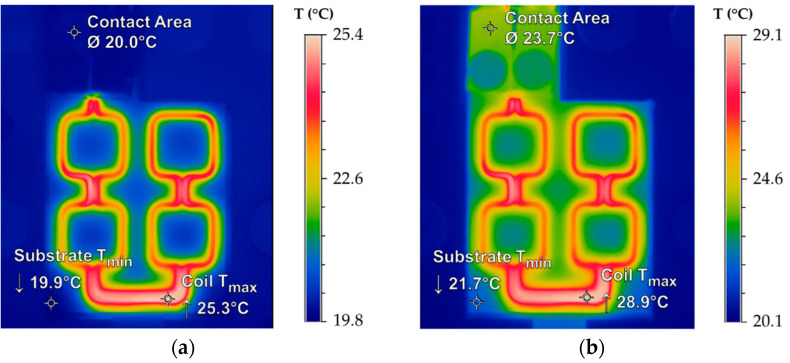
IR images of the coil self-heating for *I*_0_ = 51.8 A and *f*_0_ = 1.958 MHz: (**a**) Temperature input after *t_h_* = 1 s; (**b**) Temperature input after *t_h_* = 10 s.

For the heating experiments, the maximum generator control voltage *U_max_* = 880 V was varied in the range of *U* = 44 V to 70.4 V. This corresponds to an applied induction power of *P* = 500 W to 800 W and a measured coil current of *I*_0_ = 51.8 A to 93.6 A, respectively. Based on the determined coil inductance of *L* = 124 nH and the used inverter capacitance of *C* = 40 nF, the resonance frequency *f*_0_ of the LC resonant circuit is approximately 1.958 MHz. Related to (3), a calculated skin depth of *δ_Cu_* = 47.96 μm for copper and *δ_Sn_* = 119.27 μm for tin results. Due to the application of similar electrical parameters, the experimental and simulated results could be compared. The coupling distance was determined by the used glass substrate with a thickness of *h_G_* = 0.5 mm. The experiments were performed in two stages. In the first step, only the self-heating of the coil conductors was investigated. In the second step, the thermal management of the entire setup was analyzed based on the inductive heating of the bond frames. Figure 19 illustrates the IR thermography images of the coil self-heating after *t_h_* = 1 s (a) and *t_h_* = 10 s (b). 

The results demonstrate that the heat distribution in the micro coil is comparable to the FEM (Figure 17). With maximum temperatures in the conductors of *T_max__*_1_ = 25.3 °C after one second and *T_max__*_10_ = 28.9 °C after ten seconds, a slightly increased heating was observed. Nevertheless, the self-heating of the micro coil can be controlled very efficiently. Moreover, the influence of a stronger cooling effect on the inlet side of the cooling channel could not be observed here, indicating a more homogeneous temperature distribution.

**Figure 20:**
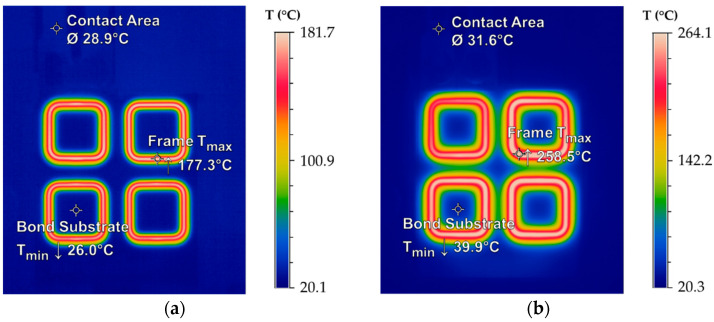
IR images of the localized inductive Cu-Sn heating for *I*_0_ = 51.8 A and *f*_0_ = 1.958 MHz: (**a**) Temperature distribution after *t_h_* = 1 s; (**b**) Temperature distribution after *t_h_* = 10 s.

Analogous to the simulation results (Figure 18), the IR images in Figure 20 show an almost homogeneous and localized frame heating after *t_h_* = 1 s (a) and *t_h_* = 10 s (b). Therefore, the electrically nonconductive glass substrate is only slightly thermally stressed. Temperature peaks at the inner corners of the frames were detected after *t_h_* = 10 s. Due to the highly selective heating, the temperature in the center of the frames can be dissipated less efficiently compared to the outer sides of the frames. As a result, the frames influence each other and cause a hot spot in the center. This proximity effect will homogenize with increasing frame number. The maximum temperature inhomogeneity, based on all heated bond frames, was found to be Δ*T*__1_ = 7 K after *t_h_* = 1 s and Δ*T___*_10_ = 32 K after *t_h_* = 10 s. The maximum heating rate was *dT*/*dt* ≈ 180 K/s with temperature peaks of *T_max__*_1_ = 177.3 °C after *t_h_* = 1 s and *T_max__*_10_ = 258.5 °C after *t_h_* = 10 s.

**Figure 21:**
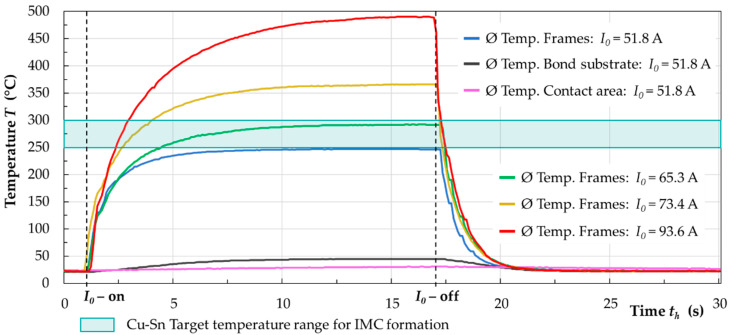
Temperature-time curves the bond substrate, the contact area, and the inductively heated bond frames for currents in the range of *I*_0_ = 51.8 A to 93.6 A and a time range of *t_h_* = 0 s to 30 s.

Figure 21 shows a comparison of the average temperatures of the bond frames, the bond substrate, and the contact area for *I*_0_ = 51.8 A with respect to the heating time *t_h_*. Additionally, the heat input into the bond frames is shown for increasing coil currents at *I*_0_ = 65.3 A, *I*_0_ = 73.4 A, and *I*_0_ = 93.6 A. 

The results show that the temperatures in the bond frames increase continuously with rising current and time. After *t_h_* = 10 s, a temperature plateau is reached for all coil currents. Consequently, a current of about *I*_0_ = 65 A with a heating time of *t_h_* = 5 s is necessary to reach the minimum target temperature of 250 °C for the formation of the intermetallic phases in the Cu-Sn binary system. After interrupting the coil current *I*_0_, the frames cool down to almost room temperature in Δ*t* ≈ 5 s. Due to the water-cooled bonding module, convection (4) and conduction (5) play a dominant role in the heat dissipation.

### 4.4. Inductive Bonding

**Figure 22:**
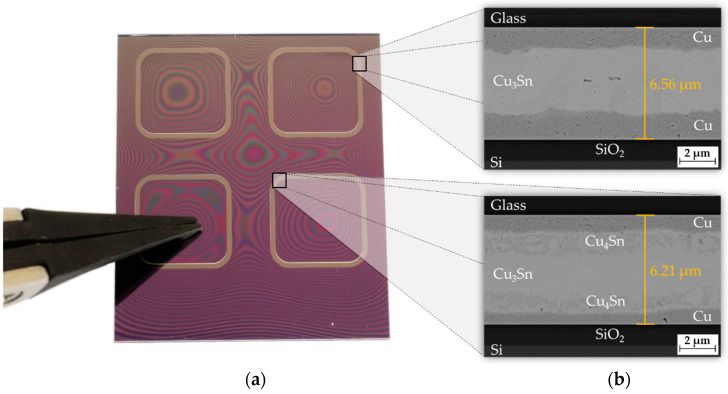
Inductive Cu-Sn SLID bonding for *I*_0_ = 78.6 A and *f*_0_ = 1.898 MHz: (**a**) Silicon-glass chip stack with Newton’s rings; (**b**) SEM images of the formed bond with the IMCs Cu_3_Sn and Cu_4_Sn.

The bond process was performed using the inductive bonding system (Figure 15). Figure 22a shows a heterogeneous substrate stack consisting of silicon and glass after inductive Cu-Sn SLID bonding. For the process preparation, the inductive bonding module was used to place the silicon and glass chips with the Cu-Sn frames on top of each other. For this, the bracket of the module served as a mechanical alignment guidance to adjust the two substrates to each other. After positioning the bond chips, a tool force of *F* = 210 N was applied, corresponding to an effective bond pressure of *p* = 3 MPa in the frames. A coil current of *I*_0_ = 78.6 A with a frequency of *f*_0_ = 1.898 MHz was applied to initiate the diffusion process. The current and frequency deviated slightly, compared to the values from the inductive heating experiments (Section 4.3), since the impedance of the entire system changed due to additional components (e.g., adapter and pressure plates). A heating time of *t_h_* = 120 s was used for the diffusion process. The tool pressure was kept for ten more seconds after the coil current was switched off to ensure a complete phase transformation. After a bonding time of *t_b_* = 130 s, the pressure plate was retracted (*p* = 0 MPa) and the bonded substrate stack could be extracted.

By applying a low pressure within a bonded frame, it could be demonstrated that the resulting interferogram with light and dark lines (Newton’s rings) could not propagate outward. This indicated a sealed bond connection. The formed IMCs are shown in Figure 22b. A complete formation to the thermodynamically stable ε-phase Cu_3_Sn is observed at the outer corner of a bonded frame. At the inner frame corner, the ε-phase is enclosed by an additionally formed phase. Ramm et al. stated that significantly above 350 °C, a further reaction within the alloy may lead to the formation of Cu_4_Sn [4]. This correlates with the observations in Figure 20, where the temperature input was higher in the inner frame corners due to the proximity effect. The interface thickness after bonding was in the range of 6 µm to 6.5 µm, which means a compression of about 0.5 μm to 1 µm. 

**Figure 23:**
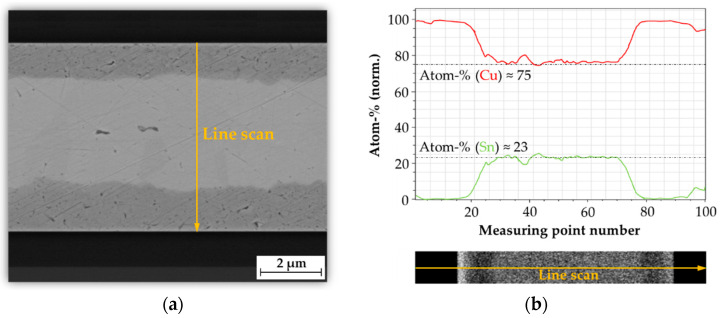
EDX analysis of the formed ε-phase Cu_3_Sn: (**a**) Position of the line scan; (**b**) Atomic percentage as a function of the phase length for Cu and Sn along the line scan.

Energy-dispersive X-ray spectroscopy (EDX) analysis was performed to allow a more detailed investigation of the formed phases. Thus, the exact compositions of the IMCs could be determined by analyzing the atomic proportion of Cu and Sn. Figure 23 shows an EDX line scan through the bonded interface as well as the atomic percentage in dependence of the phase length for the ε-phase Cu_3_Sn. Within the phase boundaries, a copper content of about *m_Cu_* = 75 % and a tin content of *m_Sn_* = 23% were found. Thus, the copper content is approximately 3 times higher than the tin content, which corresponds to a complete conversion to Cu_3_Sn.

**Figure 24:**
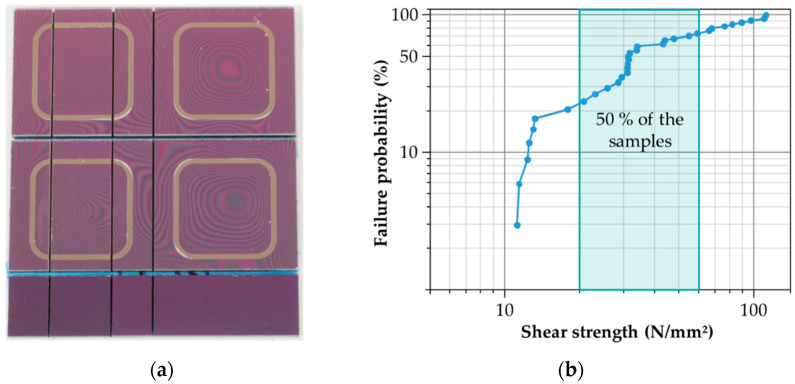
Characterization of mechanical strength: (**a**) Silicon-glass chip stack after dicing; (**b**) Shear strength of silicon-glass chip stacks bonded at *I*_0_ = 78.6 A, *f*_0_ = 1.898 MHz, *t_b_* = 130 s, and *p* = 3 MPa.

To evaluate the mechanical strength of the bonded Cu-Sn interface, the compression shear test [54] was applied. In order to ensure a concentrated force load without damaging the substrate materials due to the compression effect, the specimen geometry was adapted to the test setup. For this purpose, the bonded chip stacks were separated into different sized samples using blade dicing to reduce the shear area *A_shear_* (Figure 24a). With a dicing yield of *Y* = 100%, a quantitative indication of the mechanical strength could be obtained. The used test setup includes the material testing machine TIRAtest 2805 (TIRA GMBH, Schalkau, Germany) with an integrated shear fixture. The shear force *F_shear_* was determined by means of a load cell, considering the shear angle. With the pre-defined lateral shear area *A_shear_*, the shear strength *τ_shear_* could be calculated with (13).

(13) $$\tau_{shear}=\frac{F_{shear}}{A_{shear}}$$

Based on the aforementioned parameter set for the inductive bonding experiments, the shear strength of 34 samples was determined (Figure 24b). For this, an average shear strength of *τ_shear_Ø_* = 45.1 N/mm^2^ was obtained. The maximum shear strength for no sample failure was *τ_shear_*__0%_ = 11.2 N/mm^2^, whereas the median shear strength (50% failure) was *τ_shear_*__50%_ = 31.3 N/mm^2^. The maximum strength was measured at *τ_shear_*__100%_ = 111.8 N/mm^2^. For more than 50% of the samples, the shear strength reached values between 20 N/mm^2^ and 60 N/mm^2^. Thus, the average shear strength is in the range of conventional Cu-Sn SLID bonding [55] and significantly higher than the mechanical strength of well-known bonding methods such as adhesive and glass frit bonding (30 N/mm^2^) [54]. By further optimization of the bonding parameters, the variation of the measured shear strength can be minimized. 

## 5. Conclusions

The process sequence presented in this paper combines the advantages of SLID bonding (e.g., moderate process temperature, high operation temperature, and mechanically strong interface) with the characteristics of inductive thin film heating (e.g., very high heating rate, selective heating). To establish industrial induction-based process flows for chip and wafer bonding, the miniaturization of the induction coil is essential. The application of a micro coil with electrically conductive coil conductors embedded in a ceramic matrix for inductive heating of metal layers with thicknesses below 5 µm is demonstrated in this paper for the first time. Therefore, an efficient planar technology consisting of laser structuring and electroplating was elaborated for the coil manufacturing. Due to the high selectivity of the heating process, it could be shown that the majority of the transferred energy converts into localized heating of the bond frames, resulting in a minimized thermal load on the substrates. Thus, very high heating rates of about 180 K/s could be achieved. In contrast, a rapid cooling close to room temperature was realized in approximately 5 s, due to the large surface to volume ratio of the bond frames and the efficient heat dissipation of the inductive bonding module. With respect to the Cu-Sn binary system, the target phase Cu_3_Sn was achieved in a process time of 130 s with a tool pressure of 3 MPa. Compared to conventional Cu-Sn SLID bonding, this means a reduction of the bonding time by approximately 60 to 90% [14,15,19].

The presented inductive bonding approach based on micro coils represents a potentially highly economical and time- and energy-efficient alternative to conventional bonding processes using conduction based global heating (e.g., SLID bonding, thermo-compression bonding, eutectic bonding). The developed processing routes and equipment successfully demonstrate the integrability of induction heating into complex bonding procedures. Moreover, the unique characteristics of induction heating, especially rapid and local temperature input, will offer new opportunities in terms of the application spectrum, new material combinations, and layouts.

In future research, the presented micro coil approach will be extended by scaling the setup to wafer-level in combination with further miniaturization of the coil conductors. In addition, alternative coil materials (e.g., silver, electrically conductive ceramics) are to be investigated and used. The implementation of impedance-controlled matching networks is also planned to further increase the process efficiency by higher frequencies and the associated reduction in skin depth.

## Figures and Tables

**Table 1:** Dependence of the skin depth on the EM field frequency.

	Symbol	Unit	Parameter Range				
Frequency	f	MHz	1.00	1.25	1.50	1.75	2.00
Copper							
Skin depth @ 25 °C	*δ*	µm	67.26	60.16	54.92	50.84	47.56
Tin							
Skin depth @ 25 °C	*δ*	µm	166.9	149.28	136.27	126.16	118.02

**Table 2:** Material and design parameters of the bond substrates.

	Symbol ^1^	Unit	Parameter
**Dimensions**			
Length	*l*	mm	33
Width	*w*	mm	28
**Silicon substrate**			
Thickness	*h_Si_*	µm	675
Thermal conductivity	*λ_Si_*	W/(m·K)	153 [49,50]
Volume resistivity	*ρ_Si_*	Ω·cm	10 to 20
Thickness of thermal barrier (SiO_2_)	*h_SiO2_*	µm	2
**Borofloat^®^ 33 substrate**			
Thickness	*h_G_*	µm	500
Thermal conductivity	*λ_G_*	W/(m·K)	1.2 [51]
Volume resistivity	*ρ_G_*	Ω·cm	1 × 10^8^ (at 250 °C) [51]

^1^ Symbolism according to Figure 3 and Figure 4.

**Table 3:** Material and design parameters of the bond frames.

	Symbol ^1^	Unit	Parameter
Length	*l_f_*	mm	10
Width	*w_f_*	mm	10
Number of frames	*#*	–	4
Thickness copper	*h_Cu_*	µm	2.5
Thickness tin	*h_Sn_*	µm	1.0
Lateral frame distance	*d_f_*	mm	4
Lateral frame width	*x_f_*	µm	500
Frame radius	*r_f_*	mm	1.5
Total frame area	*A_f_*	mm^2^	1469.2

^1^ Symbolism according to Figure 3 and Figure 4.

**Table 4:** Substrate and material parameter of CeramTec Alunit^®^ 170 C.

	Symbol	Unit	Parameter
**Substrate parameter**			
Length	*l*	mm	48
Width	*w*	mm	28
Thickness	*h*	mm	1
Surface quality	–	–	as fired
Surface roughness	*R_a_max_*	µm	0.6
Flatness	$\bar{x}D$	%	0.3
**Material parameter**			
Thermal conductivity	*λ*	W/(m·K)	170 [52]
Specific heat capacity	*c_p_*	J/(kg·K)	720 @ 100 °C [52]
Volume resistivity	*ρ*	Ω·cm	1 × 10^14^ @ 20 °C [52]
Dielectric strength	*κ*	kV/mm	15 @ 0.635 mm [52]

## Data Availability

Not applicable.
